# Protein Kinase C-Dependent Dephosphorylation of Tyrosine Hydroxylase Requires the B56δ Heterotrimeric Form of Protein Phosphatase 2A

**DOI:** 10.1371/journal.pone.0026292

**Published:** 2011-10-21

**Authors:** Jung-Hyuck Ahn, Yong Kim, Hee-Sun Kim, Paul Greengard, Angus C. Nairn

**Affiliations:** 1 Department of Biochemistry, Ewha Womans University School of Medicine, Seoul, Korea; 2 Laboratory of Molecular and Cellular Neuroscience, The Rockefeller University, New York, New York, United States of America; 3 Department of Molecular Medicine and Tissue Injury Defense Research Center, Ewha Womans University, School of Medicine, Seoul, Korea; 4 Department of Psychiatry, Yale University School of Medicine, New Haven, Connecticut, United States of America; Consejo Superior de Investigaciones Cientificas, Spain

## Abstract

Tyrosine hydroxylase, which plays a critical role in regulation of dopamine synthesis, is known to be controlled by phosphorylation at several critical sites. One of these sites, Ser40, is phosphorylated by a number of protein kinases, including protein kinase A. The major protein phosphatase that dephosphorylates Ser40 is protein phosphatase-2A (PP2A). A recent study has also linked protein kinase C to the dephosphorylation of Ser40 [1], but the mechanism is unclear. PP2A isoforms are comprised of catalytic, scaffold, and regulatory subunits, the regulatory B subunits being able to influence cellular localization and substrate selection. In the current study, we find that protein kinase C is able to phosphorylate a key regulatory site in the B56δ subunit leading to activation of PP2A. In turn, activation of the B56δ-containing heterotrimeric form of PP2A is responsible for enhanced dephosphorylation of Ser40 of tyrosine hydroylase in response to stimulation of PKC. In support of this mechanism, down-regulation of B56δ expression in N27 cells using RNAi was found to increase dopamine synthesis. Together these studies reveal molecular details of how protein kinase C is linked to reduced tyrosine hydroxylase activity via control of PP2A, and also add to the complexity of protein kinase/protein phosphatase interactions.

## Introduction

Tyrosine hydroxylase (TH) is the rate-limiting enzyme involved in the synthesis of catecholamines such as dopamine [2]. The activity of TH is controlled by phosphorylation of multiple sites, including Ser8, Ser19, Ser31 and Ser40, that is catalyzed by several different protein kinases, including protein kinase A (PKA), protein kinase C (PKC), calcium/calmodulin-dependent protein kinase II (CaMKII), and the MAP kinase, ERK [3]–[5]. In particular, phosphorylation of Ser40 by PKA has been found to play a critical role in activation of TH, and has been the subject of extensive study in dopaminergic neurons and other types of cell. Elucidation of the mechanisms involved in control of TH is essential for understanding its role in the normal function of dopaminergic neurons as well as in neurodegenerative diseases such as Parkinson's disease, where dopamine synthesis is impaired.

The dephosphorylation of TH has also been the subject of a number of studies. The major protein phosphatase that dephosphorylates Ser40 of TH is believed to be protein phosphatase 2A (PP2A) based on in vitro studies as well as in studies in intact cell systems using PP2A inhibitors [6]–[8]. A recent study has linked the δ isoform of PKC to enhanced PP2A activity and reduced TH activity through dephosphorylation of Ser40 [1]. However, the detailed mechanism is not known. PP2A is ubiquitously expressed in eukaryotic cells where it exists as a heterotrimeric enzyme composed of a 36 kDa catalytic C subunit, a 64 kDa scaffolding A subunit, and multiple regulatory B subunits that are thought to influence enzyme activity, substrate specificity and subcellular localization [9]–[14]. We have recently found that the B56δ subunit is phosphorylated by PKA at Ser566 leading to activation of PP2A, and enhanced dephosphorylation of certain sites in DARPP-32 [15]–[16], a key mediator of dopamine action in striatal medium spiny neurons [17]. Other recent studies suggest that the regulation of the B56δ-containing heterotrimeric form of PP2A by PKA is not limited to medium spiny neurons and that therefore the control of protein dephosphorylation by cAMP/PKA/B56δ/PP2A may be a more widespread phenomenon [18]–[20]. In the current study, we have found that PKC phosphorylates the B56δ subunit at Ser566. Moreover, we find that the regulation of B56δ by PKC plays an important role in activation of PP2A, which in turn is responsible for enhanced dephosphorylation of Ser40 and inactivation of TH.

## Materials and Methods

### Chemicals and antibodies

Rottlerin and phorbol–12-myristate-13-acetate (PMA) were obtained from Calbiochem (La Jolla, CA) Mouse tyrosine hydroxylase, phospho-Ser40 and phospho-Ser31 antibodies, were obtained from Chemicon (Temecula, CA). Anti-FLAG antibody, benzoase, and heparin Type I pre-packed columns were obtained from Sigma-Aldrich (St. Louis, MO). Antibody to B56δ and to the various phosphorylation sites in B56δ were prepared as described [15]. Deuterated 2-(3,4-dihydroxyphenyl)ethyl-1,1-d2-amine HCl (deuterated dopamine HCl) was obtained from CDN isotope INC (Quebec, Canada).

### Cell culture

Neuro-2a (N2a) cells were purchased from ATCC (Manassas, VA), and cultured in 50% Opti-MEM and 50% DMEM containing 10% fetal bovine serum, 50 U penicillin, and 50 µg/ml streptomycin. N27 cells were grown in RPMI 1640 medium with 2 mM L-glutamine, 10% fetal bovine serum, 50 U penicillin, and 50 µg/ml streptomycin.

### Transfection

N2a cells were cultured to 60–70% confluence in 50% Opti-MEM and 50% DMEM containing 10% fetal bovine serum without penicillin-streptomycin. Expression plasmids were transfected into N2a cells in six-well plate using Fugene 6 reagent (Roche). Media were replaced with fresh media containing 50 U penicillin and 50 µg/ml streptomycin 12 h post-transfection. Forty-eight hours after transfection, cells were treated with DMSO vehicle or the indicated reagents for the indicated times. Cells were then lysed in 200 µl of a buffer containing 50 mM Tris pH 8.0, 150 mM NaCl, 1% Triton X-100, 0.5% sodium deoxycholate, 0.1% SDS, protease inhibitor cocktail (Roche), and phosphatase inhibitor cocktail (Calbiochem), followed by brief sonication and centrifugation at 10,000× g. Supernatants were used for immunoblotting as described below.

### Immunoblotting

Cell lysates were separated by 4–20% SDS-PAGE with pre-made Tris-glycine gels (Invitrogen) and immunoblotted onto polyvinylidene diflouride (PVDF) membranes (Immobilon-P; Millipore, Bedford, Massachusetts). The membranes were blocked for 2 h at room temperature in Superblock reagent (Pierce) and phosphatase inhibitor cocktail (1 ml in 100 ml of blocking reagent). Membranes were probed with the indicated antibodies. Antibody binding was detected using horseradish peroxidase-linked IgG (1∶10000; Pierce) and the ECL immunoblotting detection system (GE Healthcare Life Science). For each experiment, values for levels of phospho-proteins were corrected for loading of total protein and then data was calculated relative to the value for the control in each experiment. Normalized data from multiple experiments were expressed as means as described in the Figure legends.

### Immunoprecipitation and in vitro PKC phosphorylation assays

FLAG-B56δ wt or FLAG-B56δ S566A mutant were expressed in N2a cells and active heterotrimeric complexes were immunoprecipitated from cell lysates using anti-FLAG antibody as described [15], [21]. PKC was purified from rat brain as described [22]. Immunoprecipitated PP2A was mixed with purified PKC (100 ng) without (control) or with PKC activators (0.1 mg/ml phosphatidylserine, 0.02 mg/ml diacylglycerol, 0.2 mM CaCl_2_) and Mg ATP for 10 min in kinase reaction buffer (20 mM Tris-HCl, pH 7.4, 10 mM MgCl_2_, 150 mM NaCl).

### Viral production and purification of adeno-associated virus-RNAi (AAV-RNAi)

B56δ oligonucleotides for RNAi production, GATCCCCCCAGTGCTGTGTCCTCTTT CTTCCTGTCA AAAGAGGACACAGCACTGGTTTTTTGGAAT and CTAGATTCCAAAAAACCAGTGCTGTGTCCTCTTTTGACAGGAAG AAAGAGGACACAGCACTGG GGG, were hybridized in buffer [100 mM Tris-HCl (pH 8.0), 10 mM MgCl_2_, 150 mM NaCl], and then ligated into pAAV-H1 precut with Bgl II and Xba I. HEK293 cells (ATCC, Manassas, VA) were grown in ten 150×25-mm dishes and co-transfected with pAAV-H1/B56δRNAi or pAAV-H1 (control AAV-RNAi) and helper plasmid using calcium phosphate. Cells were trypsinized, pelleted and resuspended in buffer [0.15 M NaCl and 50 mM Tris-HCl (pH 8.0)] 72-hour post-transfection. Virus was purified using a published protocol [23] with some modifications. Briefly, after three freeze-thaw cycles (liquid nitrogen to 37°C) to lyse the cells, benzoase was added (50 U/ml, final) and the cell lysate was incubated at 37°C for 30 min. The lysate was added to a centrifuge tube containing a 15%, 25%, 40% and 60% iodixanol step gradient. Samples were centrifuged at 350,000 *g* for 60 min at 18°C, the 40% fraction was collected, then added to an affinity column containing heparin type 1 (Sigma), washed with 0.1 M NaCl and eluted with 1 M NaCl. Elution buffer was exchanged with 25 volumes of PBS using an Amicon Ultra 100K NMWL centrifugal filter. The virus was titered using an AAV ELISA kit (Progen) and kept at −80°C until time of use. AAV (1×10^12^ pfu) was added to each well of a 6-well plate (cells were typically 70% confluent) for 48 hours. AAV without any RNAi oligonucleotide was used as a control.

### Dopamine measurement

N27 cells expressing either control RNAi or B56δ RNAi were lysed and sonicated in 50 mM ammonium bicarbonate buffer (pH 8.0) after 72 h. Dopamine was measured using LC-MS/MS. Dopamine-1,1,2,2-d4 hydrochloride (CDN isotopes, Quebec, Canada) was used as a standard. The dopamine isotope was spiked into the samples and analyzed by nanoHPLC-MS with a QSTAR QqTOF mass spectrometer (Applied Biosystems).

## Results

### The PKC activator PMA increases phosphorylation of the B56δ subunit of PP2A at Ser566

**Figure 1:**
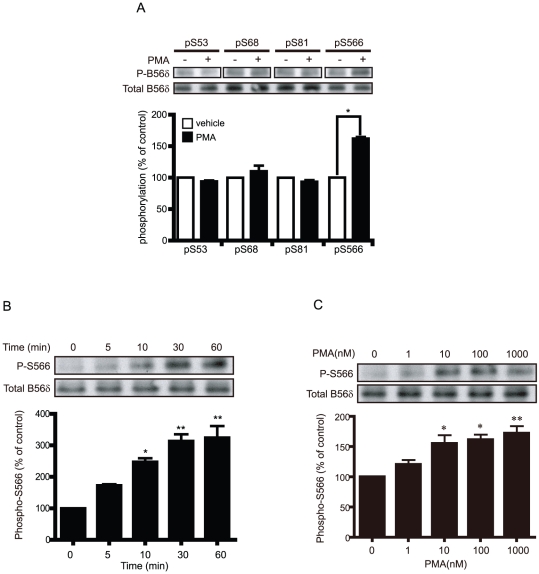
The PKC activator PMA increases phosphorylation of B56δ at Ser566. (A) N2a cells expressing FLAG-B56δ subunit were treated with DMSO (vehicle) or PMA (5 nM) for 5 min. Cells were lysed and proteins were analyzed by SDS-PAGE and immunoblotting with phospho-specific antibodies to the indicated sites in B56δ. Total B56δ was analyzed with anti-FLAG antibody. Upper panels show immunoblots. The bar graph shows quantification of immunoblot data normalized in each experiment to vehicle for each site as means ± s.e.m. (n = 3). *, P<0.001 compared with vehicle-treated control by student's t-test. (B) N2a cells expressing FLAG-B56δ were treated with different concentrations of PMA (0, 1, 10, 100, 1000 nM) for 5 min. Phosphorylation of Ser566 and total B56δ was assayed as in (A). The bar graph shows quantification of data normalized in each experiment to the zero PMA condition as means ± s.e.m. (n = 3). *, P<0.05, **, P<0.01 compared with vehicle-treated control by student's t-test. (C) N2a cells expressing FLAG-B56δ were treated with 5 nM PMA for the indicated times. Phosphorylation of Ser566 and total B56δ was assayed as in (A). The bar graph shows quantification of data normalized in each experiment to the zero time condition as means ± s.e.m. (n = 3). *, P<0.01, **, P<0.001 compared with vehicle treated control by student's t-test.

To initially address the ability of PKC to regulate the phosphorylation of the B56δ subunit, we expressed FLAG-B56δ in N2a cells, then treated cells with vehicle or the phorbol ester PMA under a variety of conditions (Figure 1). The phosphorylation level at four sites in B56δ (Ser53, Ser68, Ser81, Ser566) was analyzed with their respective phospho-specific antibody. PMA treatment increased the phosphorylation level of Ser566 ∼1.5-fold compared to vehicle-treated cells (Figure 1a). Exposure of N2a cells to different concentrations of PMA resulted in a dose responsive increase in Ser566 phosphorylation up to 10 nM (Figure 1b). In response to treatment with PMA, phosphorylation of B56δ was rapid and maximal after 30 min (Figure 1c). In the presence of 5 nM PMA, the phosphorylation at Ser566 increased ∼3.5-fold after 30 min compared to the vehicle-treated control.

### PKCδ is involved in PMA-dependent phosphorylation of B56δ

**Figure 2:**
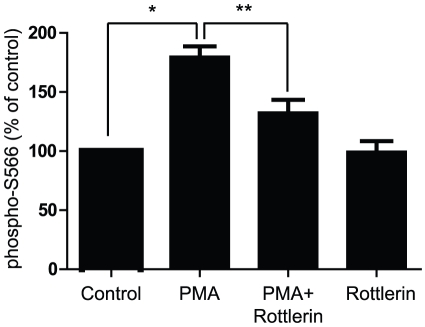
Rottlerin inhibits PMA-dependent phosphorylation of Ser566 in B56δ. N2a cells expressing FLAG-B56δ were pretreated without or with rottlerin (5 µM) for 30 min as indicated and then treated with DMSO (Control) or PMA (10 nM) for an additional 10 min. Phosphorylation of Ser566 and total B56δ was assayed as above. The bar graph shows quantification of immunoblot data (not shown) normalized in each experiment to control as means ± s.e.m. (n = 3). *, P<0.001, **, P<0.01 compared with vehicle-treated vector control by one-way ANOVA with Newman-Keuls multiple comparison test.

We next investigated the type of PKC isozyme involved in B56δ phosphorylation. FLAG-B56δ was expressed in N2a cells, and the effect of the PKCδ inhibitor rottlerin was examined (Figure 2). Cells were pre-incubated in the absence or presence of rottlerin for 30 min, treated with DMSO vehicle or PMA. PMA-dependent phosphorylation at Ser566 was inhibited by rottlerin. Rottlerin alone did not have any significant effect on Ser566 phosphorylation. Go6976, a PKCα specific inhibitor, did not affect Ser566 phosphorylation in a statistically significant manner (data not shown) suggesting that PKCδ is likely a major PKC isozyme involved in B56δ phosphorylation in N2a cells.

### PKC activates the B56δ heterotrimeric form of PP2A *in vitro* via phosphorylation at Ser566

**Figure 3:**
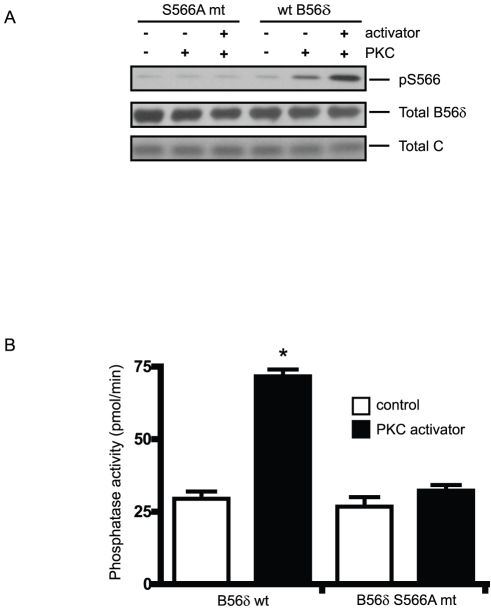
PKC activates PP2A/B56δ in vitro. FLAG-B56δ wt or FLAG-B56δ S566A mutant were expressed in N2a cells and active heterotrimeric complexes were immunoprecipitated from cell lysates using anti-FLAG antibody. Immunoprecipitated PP2A was mixed with purified PKC without (control) or with PKC activators (0.1 mg/ml phosphatidylserine, 0.02 mg/ml diacylglycerol, 0.2 mM CaCl_2_) and Mg ATP for 10 min. (A). Phosphorylation at Ser566 of B56δ was assessed by immunoblotting using anti-phospho-Ser566 antibody. As a loading control, total B56δ subunit and co-immunoprecipitated PP2A C subunit were assessed by immunoblotting. (B). Immobilized PP2A was suspended in phosphatase assay buffer then PP2A activity was measured using a phospho-peptide substrate (Promega, 5 min assay). Phosphatase activity is shown as means ± s.e.m. (n = 3). *, P<0.001, compared with no PKC activator control by student's t-test.

Our previous studies have shown that exogenous expression of the B56δ subunit results in formation of a heterotrimeric complex that can be isolated from cell lysates (Ahn *et al.* 2007a). Wild-type B56δ (FLAG-B56δ wt) or a mutant in which Ser566 was replaced by alanine (FLAG-B56δ S566A) were expressed in N2a cells, and PP2A heterotrimers were immunoprecipitated with anti-FLAG antibody. The immuno-purified PP2A preparations were then incubated *in vitro* with PKC and MgATP in the absence or presence of a PKC activator mixture which contained phosphatidylserine, diacylglycerol, and Ca^2+^ (Figure 3). Using immunoblotting, for the PP2A preparation that contained wild-type B56α subunit, a low level of phosphorylation at Ser566 was observed in the presence of PKC, and this was increased with addition of the PKC activator mixture (Figure 3A). However, as expected, only background phospho-Ser566 signal was detected in the PP2A preparation that contained B56δ with the S566A mutation.

In parallel assays, PP2A activity was measured. PKC incubation resulted in activation of the PP2A preparation containing wild-type B56δ as measured using a synthetic phospho-peptide as substrate. However, there was no effect of PKC on PP2A activity associated with the B56δ S566A mutant. We also carried out assays where ATP was replaced by [^32^P] ATP. Phosphorylation of the A, B and C subunits was detected in the absence of the PKC activator mixture, while addition of the activator mixture led to increased phosphorylation of the B56δ subunit, with a smaller increase also being observed for the C subunit, and no increase being observed for the A subunit (see supplementary Figure S1). Mutation of Ser566 prevented the increase in phosphorylation of the B56δ mutant protein, seen in the presence of the PKC activator mixture. However, mutation of Ser566 had no effect on phosphorylation of either the A or C subunits. Taken together with the phosphatase activity assays, these results indicate that phosphorylation of Ser566 in the B56δ subunit by PKC is solely responsible for the activation of PP2A activity observed in the in vitro assays.

### PP2A containing the B56δ subunit is activated by PMA, and selectively dephosphorylates Ser40 of tyrosine hydroxylase

Among several sites such as Ser8, Ser19, Ser31, and Ser40, phosphorylation of Ser40 by PKA has been found previously to be associated with increased activity of TH [5]. Interestingly, a previous study found that PKCδ was associated with activation of PP2A and dephosphorylation of Ser40 [1], but the precise mechanism was not elucidated. To address the role of PKC-mediated activation of B56δ in PP2A-mediated dephosphorylation of TH, we analyzed the phosphorylation status of Ser40 of TH in rat mesencephalic neuronal N27 cells that express significant endogenous levels of the enzyme and which produce dopamine following dibutyryl cAMP-induced differentiation [24]. Notably, N27 cells expressed higher levels of B56δ than N2a cells, and the effect of PMA on B56δ S566 phosphorylation was robust in N27 cells (data not shown).

**Figure 4:**
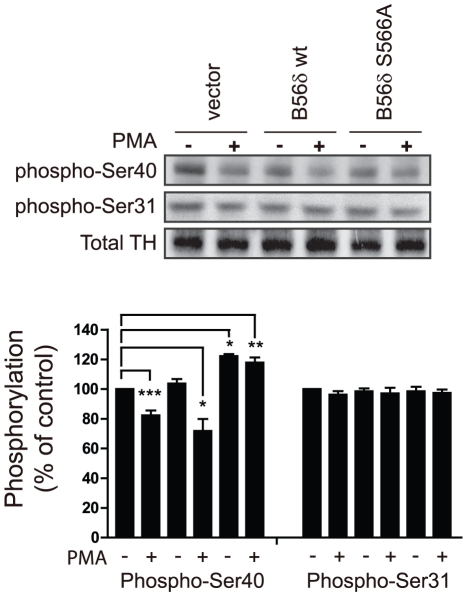
Phosphorylation of B56δ at Ser566 mediates PMA-dependent dephosphorylation of Ser40 of tyrosine hydroxylase. N27 cells expressing either vector, FLAG-B56δ wt or FLAG-B56δ S566A mutant were treated with either DMSO (-) or PMA (10 nM) for 30 min. Cells were lysed and proteins were analyzed by SDS-PAGE and immunoblotting with phospho-specific antibodies to Ser40 and Ser31 in tyrosine hydroxylase (TH), and a total tyrosine hydroxylase antibody (upper panels). The bar graph shows quantification of the immunoblot data normalized in each experiment to control as means ± s.e.m. (n = 3). *, P<0.001, **P<0.01, ***P<0.05 compared with vehicle-treated vector control by one-way ANOVA with Newman-Keuls multiple comparison test.

N27 cells expressing either vector control, FLAG-B56δ wt, or FLAG-B56δ S566A, were treated with PMA, then the phosphorylation level of Ser40 of tyrosine hydroxylase was analyzed by immunoblotting (Figure 4). In control cells (vector alone), upon PMA stimulation the phosphorylation on Ser40 was decreased. Expression of FLAG-B56δ wt resulted in a slightly larger effect of PMA but this was not statistically significant. In contrast, in cells expressing FLAG-B56δ S566A, there was an increased basal level of phosphorylation of Ser40 which was unaffected by PMA. Phosphorylation of Ser31, which was used as a control, was unaffected by any condition examined.

**Figure 5:**
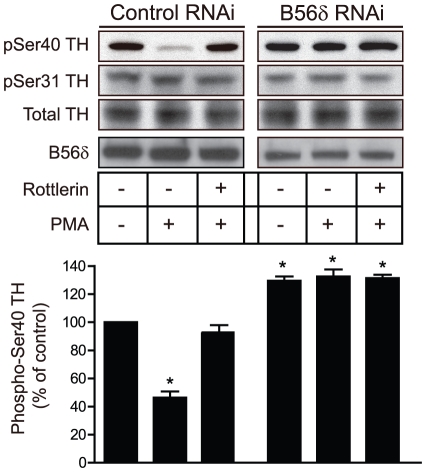
PMA-dependent dephosphorylation of Ser40 in tyrosine hyroxylase is inhibited by down-regulation of B56δ. N27 cells were infected with either AAV-control RNAi or AAV-B56δ RNAi for 48 h. N27 cells were then pretreated with either vehicle or rottlerin (5 µM) for 30 min, then treated with PMA (10 nM) for 10 min as indicated. Phosphorylation of Ser40 and Ser31 of tyrosine hyroxylase was measured as above. B56δ levels were measured by immunoblotting. The bar graph shows quantification of immunoblot data normalized in each experiment to control as means ± s.e.m. (n = 3). *, P<0.001 compared with vehicle-treated control by one-way ANOVA with Newman-Keuls multiple comparison test.

These results suggest that the FLAG-B56δ S566A mutant may be acting in a dominant-negative manner to antagonize the endogenous action of B56δ. We further examined the PKC-dependent regulation of PP2A/B56δ using RNAi to down-regulate endogenous B56δ expression in N27 cells (Figure 5). B56δ-specific RNAi, expressed via adeno-associated virus (AAV) in N27 cells, resulted in reduced expression of endogenous B56δ protein by the B56δ-specific RNAi (less than 20% compared to control RNAi). Control RNAi expression had no effect compared to non-infected cells (not shown). Control or B56δ knock-down N27 cells were treated with DMSO vehicle, PMA or PMA together with rottlerin. PMA treatment resulted in dephosphorylation of phospho-Ser40 in control RNAi-infected cells, and this was inhibited by rottlerin. However, there was no effect of PMA treatment in cells in which B56δ expression was knocked down. Notably, the basal level of phospho-Ser40 was increased following knockdown of B56δ. Phosphorylation of Ser31 was unaffected under any of the conditions used.

### PP2A containing the B56δ subunit regulates dopamine synthesis in N27 cells

**Figure 6:**
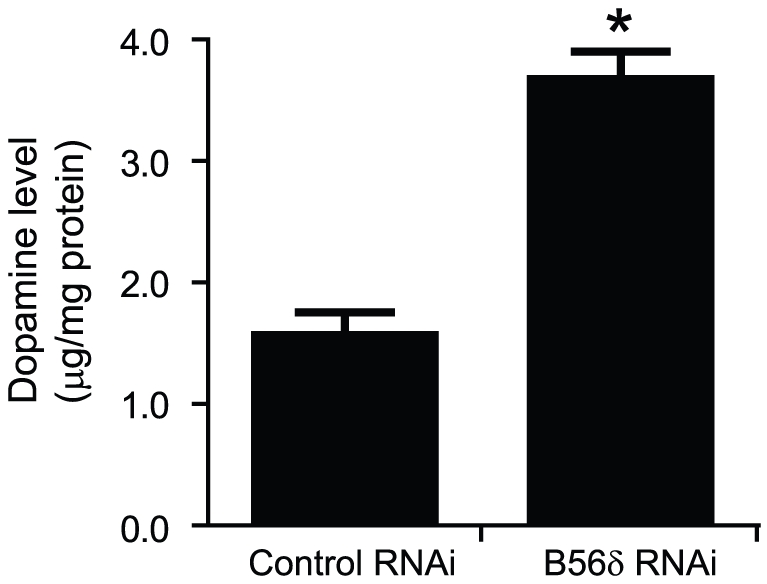
Dopamine synthesis in N27 cells is increased following down-regulation of B56δ. N27 cells were infected with AAV-control RNAi or AAV- B56δ RNAi for 72 h. Dopamine was measured in cell lysates by LC/MS with tritium labeled DA as a standard. Data are shown as means ± s.e.m. (n = 3). *, P<0.01 compared with control RNAi infected cells by student's t-test.

Previous studies have indicated that PKCδ-deficient N27 cells showed an increase in dopamine synthesis through enhanced TH activity [1]. We further examined the physiological role of TH regulation by PKC-mediated control of the B56δ subunit by measuring dopamine synthesis in N27 cells (Figure 6). Expression of B56δ was knocked down in N27 cells using AAV-mediated expression of B56δ RNAi. After 72 h, cells were harvested, and dopamine production was analyzed using LC/MS. N27 cells with reduced B56δ expression showed significantly increased dopamine levels compared to cells expressing a control RNAi plasmid. Dopamine levels were determined to be 1.59±0.16 µg/mg of protein in control RNAi cells compared to 3.69±0.21 µg/mg protein in B56δ RNAi expressing cells.

## Discussion

The results obtained in this study indicate that Ser566 in the B56δ subunit of PP2A is phosphorylated in response to activation of PKC in intact cells. Previously we have found that phosphorylation of this residue by PKA results in activation of phosphatase activity of the heterotrimeric PP2A complex containing B56δ (Ahn *et al.* 2007a). Consistent with our previous results, phosphorylation of Ser566 of B56δ was accompanied by activation of PP2A. The results obtained from studies with over-expression of wild-type and mutant B56δ, as well as of knockdown of B56δ expression, provide strong support for the conclusion that phosphorylation of B56δs causally involved in mediating the effect of PKC activation on dephosphorylation of Ser40 in TH. Our results are consistent with a role for the PKCδ isoform in regulation of the B56δ/PP2A heterotrimer based on the use of the relatively specific inhibitor, rottlerin. Various studies have found that several different isoforms of PKC are able to interact with PP2A, although nothing appears to be known about the identity of the B subunit in these complexes [25]–[28]. We therefore cannot rule out that PKC isoforms other than PKCδ might be able to regulate PP2A activity via phosphorylation of B56δ.

In previous studies, Zhang et al have shown that PKC could enhance PP2A activity resulting in dephosphorylation of Ser40 of TH [1]. Based on in vitro experiments, their study suggested that PKC could phosphorylate the C subunit of PP2A, apparently leading to increased phosphatase activity. However, the in vitro evidence for this was not strong, and the mechanism involved in intact cells was not studied. Our results indicate that B56δ phosphorylation at Ser566 is responsible for all of the effects we observed of PKC activation on TH Ser40 dephosphorylation.

A number of studies have implicated PKC in the regulation of TH in several different cellular systems [5]. However, the precise actions of PKC have been difficult to assess. In vitro, PKC is able to phosphorylate TH at Ser40 and stimulate its activity [29]. However, another in vitro study found that while PKC could phosphorylate TH at Ser40, there was no effect on activity [30]. The exact reason for the differences in these studies is not clear but may be related to the stoichiometry of phosphorylation that was achieved in the two studies. Several studies using intact cell preparations have indicated that activation of PKC leads to phosphorylation and activation of TH [31]–[33]. Some of these studies indicate that Ser40 is regulated by PKC while others suggest the likelihood that Ser31 may be the site that is targeted. Our results as well as those of Zhang et al [1] show a consistent ability of PKC activation to result in dephosphorylation of TH at Ser40, with no obvious effect at Ser31, in a number of different types of cell types in culture as well as in vivo. Presumably, the differences observed in the different studies reflect the use of various types of cells and the methods used to stimulate PKC. Based on our results, it is also possible that the cell types used in these previous studies expressed variable levels of B56δ, which has been found to have restricted cell and tissue expression [34]. In the absence of B56δ, PP2A would not be activated and therefore PKC activation might lead alternatively to phosphorylation and activation of TH.

Our recent studies have found that PP2A is subject to novel forms of regulation by second messenger-mediated signaling mechanisms. We have found that cAMP/PKA can be coupled to protein dephosphorylation via the ability of PKA to phosphorylate B56δ and activate PP2A [15]. In other studies, we have found that Ca^2+^ can activate the PR72-containing heterotrimer of PP2A [21]. The present study extends these results and shows that pathways traditionally thought to increase phosphorylation can also be coupled to pathways that result in decreased phosphorylation of selected substrates. The B subunits of PP2A likely influence the specificity of PP2A towards certain substrates through direct protein:protein interactions [10], [35]. The crystal structures of a PP2A heterotrimer containing the core domain of the B56γ isoform has provided important insight into holoenzyme assembly, and the role of the B56 subunit in substrate recognition [35]–[38]. The central core of the B56 subunit contains a number of HEAT-like repeats that make multiple contacts with the C subunit, suggesting mechanisms whereby the specificity of substrate interactions with the active site of PP2A can be influenced. A previous study found that the B56β isoform of PP2A was able to confer specificity for PP2A towards Ser40 of TH [8]. A recent follow-up study found that a specific Glu residue (Glu153) in B56β is an important molecular determinant for PP2A specificity towards TH [39]. Notably, a residue equivalent to Glu153 is found in other B56 isoforms including B56δ. The precise mechanism whereby phosphorylation of Ser566 of B56δ is able to increase PP2A activity is not known. However, phosphorylation may be able to increase PP2A affinity for TH or other selected substrates through additional protein:protein interactions, perhaps by influencing the interaction of Glu153 with specific substrates like TH.

Phosphorylation of Ser40 of TH by PKA is known to play a critical role in the regulation of enzyme activity [5]. Based on our previous studies of regulation of B56δ, one might speculate that activation of PKA could also lead to dephosphorylation of TH at Ser40. While we did not investigate this in detail, our preliminary studies suggest that stimulation of PKA results in increased Ser40 phosphorylation in the cell lines used in this study. We interpret these results and studies of TH in other cell systems as indicating that the balance between PKA-mediated phosphorylation and PKA/B56δ/PP2A-mediated dephosphorylation favors the phosphorylation of Ser40 by PKA. A similar situation is found for phosphorylation of Thr34 of DARPP-32 by PKA [15].

Our initial studies of B56δ and regulation of PP2A by cAMP/PKA-dependent signaling focused on DARPP-32, a protein that plays an important role in dopaminergic signaling in striatal neurons [16], [17]. Other more recent studies have suggested that this mechanism is more widespread and we have implicated its role in regulation of PP2A in a number of different cellular signaling processes [18]–[20]. By comparison, it seems likely that the ability of PKC to regulate PP2A via phosphorylation of B56δ may also play a role in the dephosphorylation of substrates other than TH in neuronal and non-neuronal cell types. In one study of B56δ, regulation of PP2A activity has been found to be involved in a complex signaling process in cardiac muscle involving protein:protein interactions between PP2A, PKA and the muscle A kinase anchoring protein, and the phosphodiesterase PDE4D3 [19]. This results in both feed-forward and feed-back mechanisms that are presumably designed to fine-tune cAMP signaling in muscle. A number of studies have found that PP2A interacts with various isoforms of PKC, and that in some cases PP2A is able to dephosphorylate and inactivate certain PKC isoforms including PKCδ [40]. This suggests by analogy with the example of B56δ-dependent regulation of PDE4D3 in cardiac muscle, that PKC is able to phosphorylate and activate PP2A, but that PP2A can inactivate PKC through a feed-back mechanism leading to transient control of this signaling system.

## Supporting Information

Figure S1 **PKC-mediated phosphorylation of PP2A with B56δ subunit.** N2a cells were transfected either with FLAG-B56δ S566A mutant or FLAG- B56δ wt then the lysates were immunoprecipitated using anti-FLAG antibody. Immunoprecipitated PP2A holoenzymes were eluted by adding FLAG peptide (100 µg/ml, Sigma-Aldrich, St. Louis, MO) for 10 min. Eluted PP2A with B56δ subunit complex was incubated with purified PKC and [γ-^32^P] ATP without or with PKC activator for 5 min. Immune-complexes were separated with 4-20% SDS-PAGE, and phosphorylation of PP2A subunits measured using autoradiography. After autoradiography, bands corresponding to each subunit (A, B, and C) were excised and ^32^P incorporation measured using a scintillation counter (cpm). The bar graph represents the cpm as means ± s.e.m. (n = 3). *, P<0.001, **P<0.01, ***P<0.05 compared with PKC-only treated control by student's t-test.
(EPS)
